# Changes in Neighborhood-Level Socioeconomic Disadvantage and Older Americans’ Cognitive Functioning

**DOI:** 10.1093/geroni/igaa057.1561

**Published:** 2020-12-16

**Authors:** Jason Settels, Anja Leist

**Affiliations:** University of Luxembourg, Esch-sur-Alzette, Luxembourg

## Abstract

Most studies of later life cognitive functioning have focused on individual-level variables. While some studies have examined neighborhood-level variables as influences upon older adults’ cognitive functioning, this scholarship has neglected to consider neighborhoods in a dynamic context. The present study helps fill this research gap by considering how changing extents of neighborhood-level socioeconomic disadvantage cause changes in older residents’ cognitive functioning. We employ waves 2 (2010-2011) and 3 (2015-2016) of the National Social Life, Health, and Aging Project (NSHAP) as our source of individual-level variables and the American Community Survey as our source of neighborhood-level variables. Our analytical sample includes 1,989 respondents who participated in both waves and were 50 to 90 years in age at wave 2 of the NSHAP. Through structural equation modelling, we find that rising neighborhood-level socioeconomic disadvantage significantly decreases older residents’ cognitive functioning, both without and after controlling for baseline neighborhood-level socioeconomic disadvantage and cognitive functioning. Furthermore, approximately 7.2% of this effect is mediated through decreases in the sizes of networks of close confidants, and roughly 8.5% of this effect occurs through increased depressive symptoms. Our findings suggest that older adults’ cognitive decline can be slowed down or prevented through improvements in their living environments. In particular, policies and programs that improve living spaces while also facilitating older residents’ development of close and supportive confidant ties are likely to be particularly effective. Our study encourages further research on how neighborhood dynamics affect older persons’ cognitive functioning.

